# Evidence of recombination among early-vaccination era measles virus strains

**DOI:** 10.1186/1471-2148-5-52

**Published:** 2005-10-06

**Authors:** Mikkel H Schierup, Carl H Mordhorst, Claude P Muller, Laurids S Christensen

**Affiliations:** 1 Bioinformatics Research Center (BiRC), University of Aarhus, Hoegh Guldbergs Gade 10, Building 090, DK-8000 Aarhus C, Denmark; 2 Department of Virology, Statens Serum Institut, Copenhagen, Denmark; 3 Institute of Immunology, Laboratoire National de Santé. PO Box 1102, L-1011 Luxembourg; 4 Department of Clinical Microbiology, Rigshospitalet, Copenhagen, Denmark

## Abstract

**Background:**

The advent of live-attenuated vaccines against measles virus during the 1960'ies changed the circulation dynamics of the virus. Earlier the virus was indigenous to countries worldwide, but now it is mediated by a limited number of evolutionary lineages causing sporadic outbreaks/epidemics of measles or circulating in geographically restricted endemic areas of Africa, Asia and Europe. We expect that the evolutionary dynamics of measles virus has changed from a situation where a variety of genomic variants co-circulates in an epidemic with relatively high probabilities of co-infection of the individual to a situation where a co-infection with strains from evolutionary different lineages is unlikely.

**Results:**

We performed an analysis of the partial sequences of the hemagglutinin gene of 18 measles virus strains collected in Denmark between 1965 and 1983 where vaccination was first initiated in 1987. The results were compared with those obtained with strains collected from other parts of the world after the initiation of vaccination in the given place. Intergenomic recombination among pre-/early-vaccination strains is suggested by 1) estimations of linkage disequilibrium between informative sites, 2) the decay of linkage disequilibrium with distance between informative sites and 3) a comparison of the expected number of homoplasies to the number of apparent homoplasies in the most parsimonious tree. No significant evidence of recombination could be demonstrated among strains circulating at present.

**Conclusion:**

We provide evidence that recombination can occur in measles virus and that it has had a detectable impact on sequence evolution of pre-vaccination samples. We were not able to detect recombination from present-day sequence surveys. We believe that the decreased rate of visible recombination may be explained by changed dynamics, since divergent strains do not meet very often in current epidemics that are often spawned by a single sequence type. Signs of pre-vaccination recombination events in the present-day sequences are not strong enough to be detectable.

## Background

Measles virus (MV) has a genome with negative polarity, consisting of non-segmented single-stranded RNA of approximately 15.9 kb. MV belongs to the *Paramyxoviridae *family in the order of *Mononegavirales *and only a single serotype is known. It is among the most infectious viruses known for humans, and no other host species has been identified. Only human populations of a considerable size are able to sustain circulation [1]. Global vaccination programs have resulted in a dramatic decline in measles cases and the documented discontinuation of indigenous circulation in a number of countries [2-4] has encouraged authorities to accomplish global control of measles. However, measles still cause a large number of deaths every year, mainly in developing countries, where endemic circulation of MV is still ongoing [5,6] as a result of poor vaccination coverage.

Intergenomic recombination has been documented in a vast number of virus species within most families of RNA and DNA viruses. Recombination can allow variants of a population to escape from the current fitness peak (escape from Muller's ratchet) and re-appear with a new phenotypic make-up and/or even re-establish in a new host-relationship [7,8]. However, recombination has never convincingly been documented in species of the *Mononegavirales*, and it is speculated whether this reflects an inability of these viruses to recombine. Yet, the order of *Mononegavirales *comprises groups of viruses with an apparently large evolutionary potential with frequent shifts of host species. Over the past decades, a considerable number of *Mononegavirales *members causing diseases in host species in which they had not previously been recognized have been identified. Examples are phocid distemper virus [9], Hendra virus [10], Menangele virus [11], Nipah virus [12] – not to forget the re-emerging divergent members of the *Filoviridae *(Marburg -and Ebola-like viruses) [see [13,14]]. The origins of these emerging viruses have not been identified and the mechanism(s) of their ability to explore new niches remains enigmatic.

Co-infection of host cells with phylogenetically distinct virus strains is required for recombination events to be detectable in sequence surveys. As a result of the global vaccination against measles a situation of endemic co-circulation of multiple strains [5,6,15] shifted to a situation with a limited number of strains being re-introduced to susceptible subpopulations in major geographical regions [2-4,16]. By sequencing part of the hemagglutinin gene [15], we recently characterized 18 MV strains collected during the pre-/early-vaccination era in Denmark. In the present study, the partial sequence of the hemagglutinin coding region of those older strains is subjected to further analysis and compared with strains sampled after vaccination (generally more recently identified) using various approaches to test for the presence of intergenomic recombination.

## Results and discussion

The term *pre-/early-vaccination era isolates *used for isolates collected in Denmark during the period of 1965–83 is meant to reflect that these isolates are from a period when vaccination against measles was not practiced in Denmark but was gradually becoming common practice in many other countries in the World. Thus, it cannot be excluded that vaccination in other countries influenced measles virus strains circulating in Denmark at the time, but it is anticipated that these isolates still bear valuable information on the nature of strains circulating before an influence of vaccination was imposed.

Table 1 compares differences in basic population genetics of the data sets of the pre-/early- and post-vaccination eras. As might be expected, the global post-vaccination data set shows more sequence variability than the Danish pre-/early-vaccination era data set, whereas the latter shows relatively more singletons, i.e. variants present only in a single sequence. Some of these singletons might be sequencing errors in the pre-/early-vaccination sequences caused by partial degradation as also observed in other ancient DNA studies [33]. Sequencing errors may also explain the higher proportions of transversion substitutions (changes between purine and pyremidine base pairs) and the higher dn/ds ratio in the pre-/early-vaccination data set. For these reasons, singletons are not considered in any of the recombination analyses. Informative sites (i.e. non-singletons) are considered unlikely to be artefacts of the sequencing procedure, since one would not expect the same error to occur more than once, and all informative sites in the pre-/early-vaccination sample are also informative sites in the post-vaccination sample. Codon usage bias measured as the effective number of codons (ENC) is low in both data sets.

**Table 1:** Basic population genetics summary of the data sets of the pre-vaccination and post-vaccination eras.

	Danish early-vaccination era data set	Global post-vaccination era data set
Length of sequences	800	800
Number of sequences	18	40
Average number of differences	12.2	31.0
# synonymous substitutions	26	131
# non-synonymous substitutions	32	71
Average Pi (nucleotide diversity)		
1. all sites	0.014	0.040
2. synonymous sites	0.030	0.109
3. non-synonymous sites	0.009	0.019
Transition/transversion bias	2.2	9.4
Codon usage bias (ENC)	53	55

**Figure 1:**
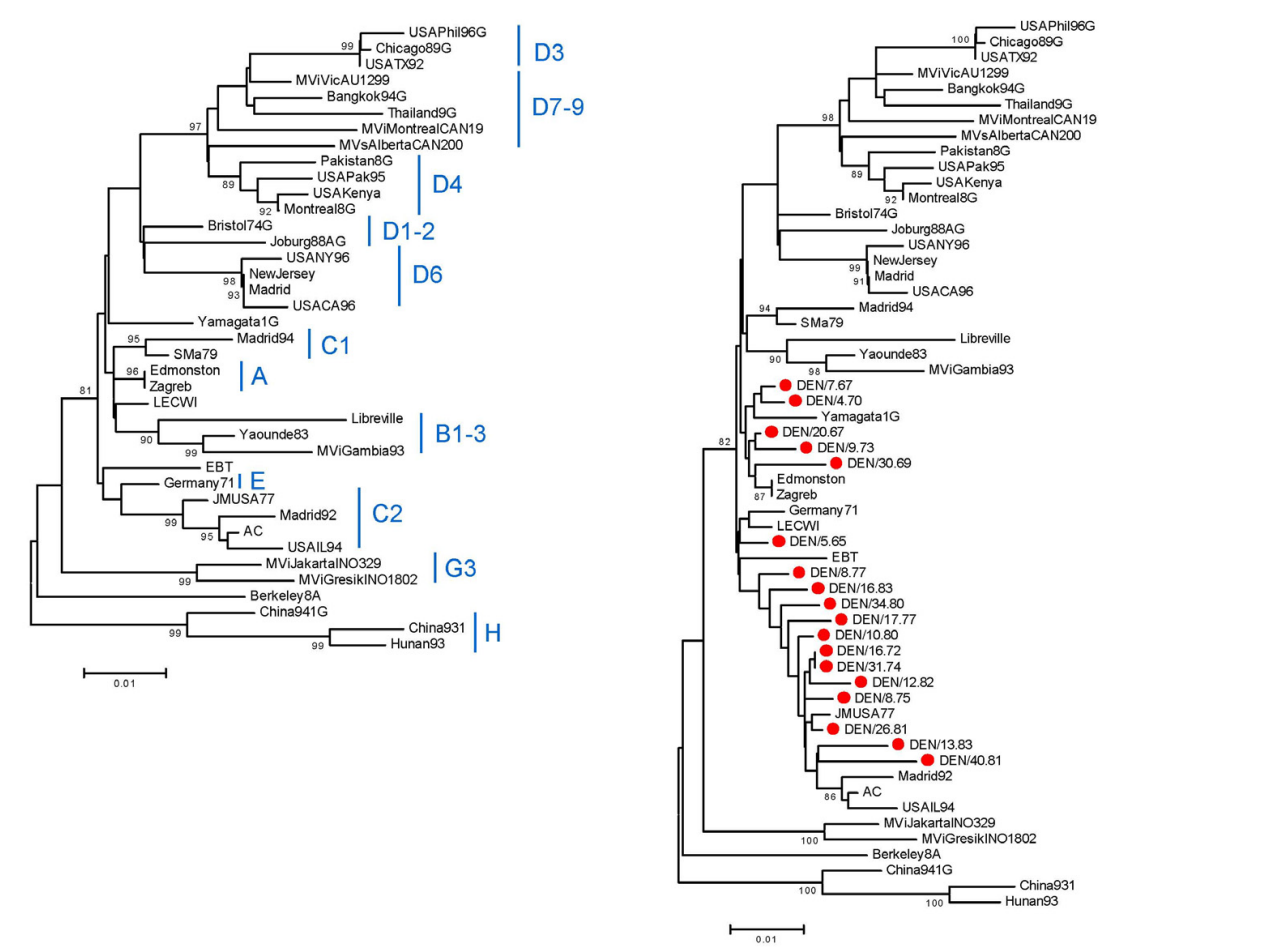
Phylogenetic trees reconstructed using the HKY substitution model and the minimum evolution criterion as implemented in MEGA 2.1 [25]. Bootstrap values >80% are shown. a) Tree based on the global post-vaccination era sample with classification of types marked, b) the same sequences as in a) plus the 18 early-vaccination era sequences, marked with a red circle.

Figure 1a shows a minimum evolution tree of the 40 post-vaccination era isolates. The tree is topologically almost identical to the one of Christensen et al. [15] and bootstrap values support many of the genome types identified at present even though the tree is based on only 800 base pairs of the hemagglutinin gene for direct comparison with the pre-/early-vaccination sample [17-19].

Figure 1b shows a tree with the same sequences as in Figure 1a, but adding the 18 pre-/early-vaccination era isolates from Denmark. As shown previously [15], the Danish isolates cluster with genome types A, C2 and E. It is also clear that the inclusion of pre-/early-vaccination era samples makes the distinction of these three genome types less obvious since the similarity distances between post-vaccination era representatives of the genome types A, C2 and E are broken down to the sum of minor differences between the pre-/early-vaccination era isolates. This might reflect that the pre-/early-vaccination era sequences are generally older than the rest and thus at the basis of division of genome types as are also the strains used for vaccine development (e.g. Edmonston). Alternatively, frequent recombination among pre-/early-vaccination era genome types at that time would lead to a poorly resolved tree. Less recombination among different surviving strains after vaccination would then lead to the more differentiated genome types seen today. The subsequent recombination analysis addresses this possibility.

### Analysis of recombination

**Figure 2:**
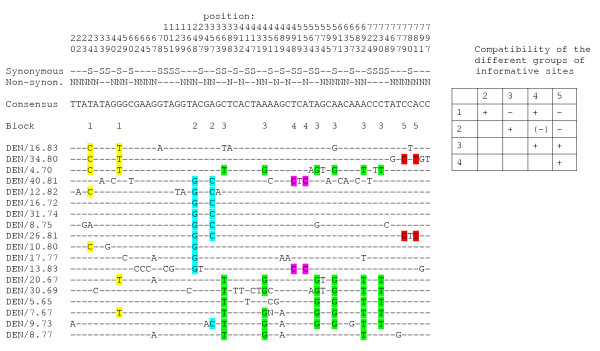
Segregating sites in the pre/early-vaccination era sequences with indication of position and state (synonymous or nonsynonymous). Different colours identify five different blocks of sites in strong LD. Table inserted indicates compatibility (+) or incompatibility (-) among blocks. Blocks 2 and 4 are only partly incompatible.

Figure 2 shows all the segregating sites for the pre-/early-vaccination era sequences with positions marked by base pair, and whether a substitution is synonymous or non-synonymous or both (for multiple hits and complex codons). It is immediately clear that many incompatibilities (by the four-gamete test [27]) exist, but also that apparent groups of physically close sites are incompatible with other such groups. We identified by eye five such groups of informative sites and marked them with different colours. Comparing these five groups by the four gamete test demonstrates that some pairs of blocks are incompatible with the same evolutionary tree (Fig. 2b). Even though the identification of these groups is subjective, the presence of such groups suggests either that they have evolved on different evolutionary trees (i.e. recombination) or, alternatively, each of the sites for a given group has mutated at least twice, i.e. by parallel mutations, at approximately the same time (since there is strong linkage disequilibrium within all groups except block 1). Note that the incompatibilities within e.g. block 1 do not weaken this conclusion. A similar table was prepared for the post-vaccination era data set. Here we were not able to visually identify groups of sites failing to satisfy the four-gamete test (results not shown), and the blocks identified in Figure 2 are not incompatible with one another in the post-vaccination data set even though it contains all of the segregating sites. Thus, if the patterns observed in Table 1 were due to convergent evolution of groups of functionally important sites in different lineages, such convergent evolution does not play an important a role in present-day evolution of measles virus. The proportion of pairs of informative sites which is incompatible by the four gamete test is 43% in the pre-/early-vaccination data set as compared to only 23% in the post-vaccination data set even though the variability in general is lower among the pre-/early-vaccination sequences.

**Figure 3:**
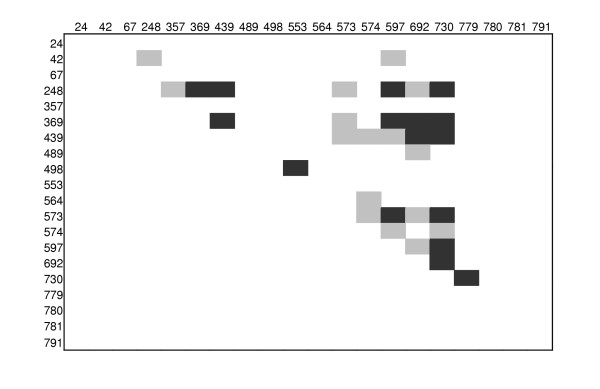
Linkage disequilibrium triangle plot for informative sites in the pre/early-vaccination sample. Significant linkage disequilibrium is indicated my shading, grey shading is P < 0.05, black shading, P < 0.001 by Fisher's exact test.

**Figure 4:**
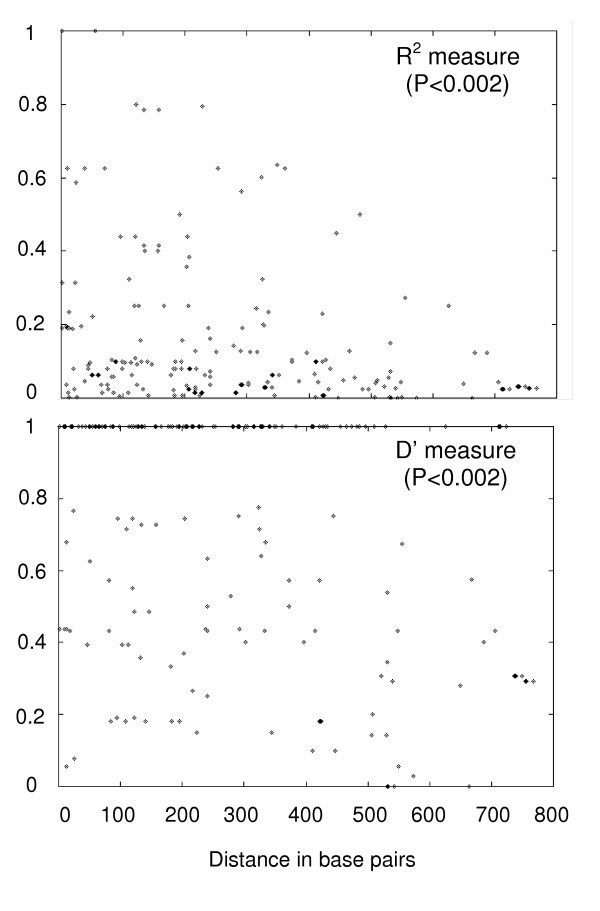
Correlation between linkage disequilibrium and distance for the early-vaccination era data set using the *R*^*2 *^and *D*' measures of LD, respectively.

One way to distinguish whether incompatibilities are caused by parallel mutations (i.e. true homoplasies) or by recombination is to correlate linkage disequilibrium (LD) between sets of informative sites to distance. Recombination causes a decrease in linkage disequilibrium and more recombination is expected between sites that are further apart from each other. The block structure of close sites is visualised in a different way in Figure 3, which shows all the significant cases of linkage disequilibrium. Visual inspection of the Figure suggests that physically close sites appear to be more likely to be in strong LD than sites far apart. This observation is tested more formally by correlating LD with distance (Table 2 and Figure 4). Table 2 shows the correlation of two commonly used measures of LD for both data sets. A significant negative correlation is observed by both measures of linkage disequilibrium in the pre-/early-vaccination data set, whereas the post-vaccination data set does not show any significant decay of linkage disequilibrium with distance. The decrease in linkage disequilibrium with distance for the pre-/early-vaccination sequences is shown graphically in Figure 4.

**Table 2:** Summary of numerical analysis of recombination in the samples of the pre-vaccination and post-vaccination eras A negative correlation implies that LD decays with distance, the P-values are obtained by a permutation test (see Methods).

	Early-vaccination sample	Post-vaccination sample
*R*^2^-correlation (P-value)	-0.26 (P < 0.01)	-0.03 (P < 0.05)
*D*' correlation (P-value)	-0.32 (P < 0.001)	0.01 (P > 0.05)
LDhat estimate of ρ (P-value)	15.8 (P < 0.002)	7.2 (P > 0.05)

An analysis of expected number of homoplasies caused by parallel mutations and the number of apparent homoplasies in the most parsimonious tree was performed on the pre-/early-vaccination data set [see [32,35]]. The basic idea is to investigate whether the number of apparent homoplasies in the most parsimonious tree is likely to be due to parallel (recurrent) mutations. If this is not the case, it is a strong indication of recombination since recombination easily creates "incompatible pairs of sites", i.e. pairs of sites that do not fit into the same phylogenetic tree, and thus will appear as homoplasies if assuming no recombination and a single phylogenetic tree. There are 800 sites. Of these, 267 are third position sites, and among these we observe 20 transition mutations. If we assume equal mutation rates at all third position sites, then the number of transition mutations we expect have happened while observing 20 different ones is 20.8+/-0.9 (calculated as the sum of geometric distributions). In the most parsimonious tree found using PAUP*, there are 26 transition mutations. In other words, given the observed number of mutation events of the transition type, we expect 0.8+/-0.9 parallel mutations/homoplasies under the assumption of no recombination (and no codon usage bias). We observe, however, 6 parallel mutations in the tree. This suggests that some of the apparent homoplasies resulted from recombination rather than from recurrent mutations. We also calculated the *effective *sites number [35] from the observed codon usage bias. However, since the codon usage bias is low, the effect is minor, and only 1.0+/-1.0 homoplasies are expected under this model, again significantly smaller than the six apparent homoplasies. A large amount of mutation rate heterogeneity at the synonymous sites offers an alternative explanation of the excess homoplasies. While this explanation cannot be ruled out, analysis of the post-vaccination data set does not support large rate heterogeneity at silent sites in the evolution of present-day measles virus, since the number of homoplasies in this data set can be explained by recurrent mutations (results not shown).

Given these different lines of evidence for recombination, it is of interest to try to estimate the rate of recombination needed to explain the data. The most appropriate method is the finite site, composite likelihood approach implemented in LDhat [31]. The result (Table 2) is that a significantly positive recombination rate is found in the pre-/early-vaccination sequences, much reflecting the results of the similar R^2 ^test. An estimated rate of ρ = 15.8 corresponds to that an expected 45 recombination events have happened in the ancestral history of the 18 pre-/early-vaccination sequences. The estimated recombination rate appears smaller than rates reported for HIV and other viruses [31]. The estimated recombination rate in the post-vaccination era data sets also shows a positive rate of ρ, but it not significantly different from zero by the permutation test (Table 2). The lack of evidence of recombination in present day sequences of MV strains is consistent with what was also observed by [31].

In conclusion, the analysis of pre-/early-vaccination era MV sequences shows evidence of recombination at rates important to the evolution of MV. The five different tests of recombination should not be considered independent tests and some of the results might be explained by alternative mechanisms such as convergent evolution of functionally important sites and rate heterogeneity of synonymous variation. However, all tests agree and provide evidence of recombination both through an excess of apparent homoplasies compared to the expected frequency of parallel mutations, and through a decrease in LD with distance, which is difficult to explain by any other hypothesis than recombination. Furthermore, it is difficult to imagine a mechanism other than recombination by which apparent homoplasies could occur pairwise or in triplets in distant parts of the sequence considering also that such patterns are not seen in the post-vaccination data set.

The evidence of recombination among pre-/early-vaccination era MV strains and the lack of detection of recombination among post-vaccination era MV strains are consistent with the shift in epidemiology from a situation of co-circulation of strains in populations to a bottleneck situation with incidental introduction of a single strain to a susceptible sub-population of a geographical region. Given that intergenomic recombination and co-infection of individuals are common phenomena of MV it might be assumed that the lineages surviving till today did emerge from a pool of recombining pre-vaccination era strains. A lower level or absence of recombination due to changed epidemiology since then has erased our ability to detect recombination in a global sample of present day MV despite its high level of variability. It is possible that the Danish pre-/early-vaccination era strains are representatives of the very pool in which recombination took place while present-day MV strains are representatives of temporally and/or geographically separated lineages. Analysing informative sites in other parts of the MV genome of present-day lineages render it unlikely that these lineages could have derived from a clonal population structure of a global pool of MV strains (L. S. Christensen, unpublished data).

The template for replication in members of the *Paramyxovirinae *is a nucleocapsid complex in which each nucleocapsid monomer (N) is predicted to be associated by hydrophobic bonds with 6 nucleotides in such a way as to resist non-ionic detergent and high salt and to protect the RNA from RNase digestion [36]. This tight association of RNA and protein has been dubbed "the rule of six" and excludes the intracellular presence of naked viral RNA molecules. It raises the question of the mechanism of RNA polymerase template recognition and has provided an explanation of the hypothesis that recombination possibly cannot occur in this group of viruses. Our data suggests that a mechanism of partial unwinding of the nucleocapsid structure exists to allow homologous intergenomic recombination or RNA polymerase template shift during replication.

## Conclusion

Measles virus appear to possess the ability to recombine but the present-day epidemiology of the virus where different sequence types rarely or never meet make the impact of recombination on the distribution of sequence diversity negligible. However, in the prevaccination area, endemic MV allowed more divergent strains to meet and recombine. The present-day strains are thus descendants of recombined sequences but the signal of the early recombination is lost in present-day sequences.

## Methods

### Sequence data

The Danish pre-/early-vaccination sequences consist of an 800 base-pair region (nt. 659 to 1458) of the hemagglutinin coding region of 18 strains collected in Denmark, Greenland and the Faeroe Islands between 1965 and 1983 (GenBank accession numbers AJ417850-AJ417867) [15]. Post-vaccination sequences of 40 strains, representative of the 22 phylogenetic clusters identified [17-19], were trimmed to match the region of the pre-/early-vaccination era sequences. GenBank accession numbers of 33 of these sequences can be found in [15]. The remaining 7 HA sequences that complete the list of globally circulating genome types, identified at present, are those of strains Mvi/Gambia/93 (Type B3, Acc. No AF484955) [20], MVi/Alberta.CAN/20.00/1 (Type D7, Acc. No. AF410986) (G.A. Tipples et al., submitted 14-Aug-2001), MVi/Montreal.CAN/19.98 (Type D8, Acc. No. AF410985) (G.A. Tipples et al., sumitted 14-Aug-2001), MVi/Vic.AU/12.99 (Type D9, Acc No. AY127853) [21], (Type G2, Acc. No. AF243851) [22], MVi/Gresik.INO/18.02 (Type G3, Acc. No. AY184218) (P.A. Rota and S.L. Liffick, submitted 19-Nov-2002), and China94-1 (Type H2, Acc. No. AF045203) [23].

### Sequence analysis

The alignment of the 18 pre-/early-vaccination and 40 post-vaccination sequences does not contain any gaps. The computer program DnaSP 3.99 [24] was used for estimation of standard parameters of population genetics. Segregating sites were classified as synonymous or non-synonymous, except for complex codons where a site may be classified as both. Phylogenetic trees from the post-vaccination data set and the two data sets combined were built using MEGA 2.1 [25] and the minimum evolution criterion. The HKY substitution model [26] was assumed. Bootstrap values were estimated from 2000 re-samples.

Recombination was examined using five different, but complementary, approaches. (i) A graphical method by which sets of sites in strong linkage disequilibrium (LD) were visually identified and marked with different colourings (Fig. 2). It was then investigated whether different sets of blocks were incompatible by the four-gamete test [27]. If blocks are incompatible, one will infer either recombination or recurrent mutation of the sites in a block at about the same time. (ii) A plot of significant pairwise linkage disequilibria was constructed using DnaSP. (iii) Decay of linkage disequilibrium with distance (measured as either the squared correlation coefficient *R*^2 ^or the standardized measure *D*') was investigated following [28] and estimated using the R2-program [29]. Analysis was restricted to informative sites. (iv) Estimation of the scaled population recombination rate ρ by a composite maximum likelihood approach [30], using the LDhat program of [31]. This program also allows a test of the null hypothesis of no recombination (ρ = 0) by a permutation test. This analysis was also restricted to informative sites. (v) Comparison of the expected number of homoplasies to the number of apparent homoplasies in the most parsimonious phylogenetic tree, closely following the approach of [32], using PAUP* [32].

## Authors' contributions

CHM, CPM and LSC collected the data. LSC formulated the hypothesis, and MHS performed all analyses. MHS and LSC wrote the paper.
